# Nanostructured Lipid Carriers Aimed to the Ocular Delivery of Mangiferin: In Vitro Evidence

**DOI:** 10.3390/pharmaceutics15030951

**Published:** 2023-03-15

**Authors:** Debora Santonocito, Ignazio Barbagallo, Alfio Distefano, Giuseppe Sferrazzo, Maria Vivero-Lopez, Maria Grazia Sarpietro, Carmelo Puglia

**Affiliations:** 1 Department of Drug and Health Sciences, University of Catania, Viale Andrea Doria n°6, 95123 Catania, Italy; 2 NANOMED-Research Center on Nanomedicine and Pharmaceutical Nanotechnology, University of Catania, 95125 Catania, Italy; 3 Department of Biomedical and Biotechnological Sciences, University of Catania, 95123 Catania, Italy; 4 Departamento de Farmacología, Farmacia y Tecnología Farmacéutica, I+D Farma (GI-1645), Facultad de Farmacia, Instituto de Materiales (iMATUS) and Health Research Institute of Santiago de Compostela (IDIS), Universidade de Santiago de Compostela, 15782 Santiago de Compostela, Spain

**Keywords:** ocular diseases, mangiferin, lipid nanoparticles, oxidative stress

## Abstract

Although mangiferin (MGN) is a natural antioxidant that could be a good candidate for the treatment of ocular diseases, its use in ophthalmology is strongly compromised due to its high lipophilicity. Its encapsulation in nanostructured lipid carriers (NLC) seems to be an interesting strategy for improving its ocular bioavailability. As reported in our previous work, MGN–NLC showed high ocular compatibility and fulfilled the nanotechnological requirements needed for ocular delivery. The aim of the present work was to investigate, in vitro and ex vivo, the capability of MGN–NLC to act as a potential drug delivery system for MGN ocular administration. The data obtained in vitro on arising retinal pigment epithelium cells (ARPE-19) did not show cytotoxic effects for blank NLC and MGN–NLC; likewise, MGN–NLC showed the maintenance of the antioxidant role of MGN by mitigating ROS (Reactive Oxygen Species) formation and GSH (glutathione) depletion induced by H_2_O_2_. In addition, the capacity of MGN-released to permeate through and accumulate into the ocular tissues was confirmed ex vivo using bovine corneas. Finally, the NLC suspension has been formulated as a freeze-dried powder using mannitol at a concentration of 3% (*w*/*v*) in order to optimize its storage for long periods of time. All this evidence suggests a potential application of MGN–NLC in the treatment of oxidative stress-related ocular diseases.

## 1. Introduction

Oxidative stress is the imbalance between the production of reactive oxygen species (ROS) and the endogenous antioxidant defense system [1]. Normally, ROS are produced in small quantities and are involved in cell signaling; then, they are reduced by the antioxidant defense systems. Glutathione (GSH) is the most abundant endogenous antioxidant of our body, and it plays a key role in protecting cells against oxidative stress-induced cellular damage [2]. Decreased GSH levels are associated with aging and a wide range of pathological conditions, including eye disorders. In fact, it has been demonstrated that GSH belongs to the defense system involved in protecting the eye against oxidative stress [3]. In pathological conditions, an accumulation of ROS is produced due to an imbalance between the excessive production of oxidizing factors (such as free radicals) and the decrease in antioxidant defenses resulting in cell damage and death [4,5]. Recent studies indicate that the normalization of antioxidant capacity could represent a very promising therapy [6] for the treatment of ocular diseases associated with marked oxidative stress, such as macular degeneration and diabetic retinopathy.

**Figure 1:**
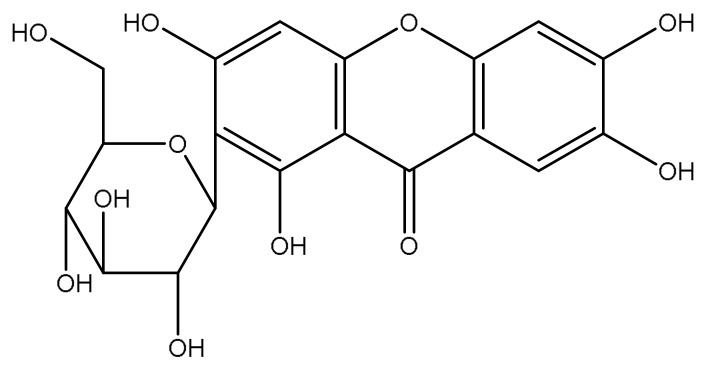
Chemical structure of MGN.

Mangiferin (MGN, 1,3,6,7-tetrahydroxy-2-[3,4,5-trihydroxy-6-(hydroxymethyl) oxan-2-yl]-9*H*-xanten-9-one) is a natural compound, mainly isolated in various parts of *Mangifera indica* L., including leaves, stem barks and fruits [7], and it possesses interesting anti-cancer, anti-inflammatory, neuroprotective, anti-diabetic and antioxidant health-related properties [8,9]. The latter is due to the hydroxyl groups present in its chemical structure (Figure 1) that are capable of preventing the production of hydroxyl radicals thanks to their iron chelating ability in Fenton-type reactions [10]. It has been shown that MGN could be a good candidate for the treatment of different oxidative stress-related ocular diseases [11]. Although MGN is a powerful antioxidant, due to its scarce solubility in water and low bioavailability, it requires specialized delivery systems that are able to entrap the active compound in an environment that is suitable for protecting it against oxidative degradation. Recently, Sguizzato and coworkers demonstrated the effectiveness of mangiferin loaded in ethosomes and transethosomes in the treatment of skin disorders related to pollutants [12]. In particular, the authors evaluated, in vitro, the antioxidant and anti-inflammatory effect of ethosomes and transethosomes on human keratinocytes exposed to cigarette smoke as an oxidative and inflammatory challenger. The results pointed out an interesting effect of the nanocarriers in modulating MGN antioxidant activity. In a recent work of our research group, we evaluated the nanostructured lipid carriers (NLC) as MGN delivery systems [11]. NLCs are a newer generation of lipid nanoparticles containing a binary mixture of solid and liquid lipids. This formulative approach applied to the lipid matrix of the nanoparticle leads to the distortion of the solid lipid crystallinity, providing higher drug payloads, less drug leakage and better colloidal stability compared to the “old” SLNs. NLCs can offer several advantages for ocular application: excellent tolerability, prolonged and controlled drug release, good corneal residence time and the biodegradability of lipids (Generally Recognized As Safe; GRAS) [13,14,15,16,17,18,19].

In our previous work [11], we elaborated the MGN–NLC formula using biocompatible and safer surfactants and lipids suitable for drug administration into the posterior eye segment. MGN–NLC showed good nanotechnological parameters which are suitable for ocular delivery and high ophthalmic tolerability. In addition, the antioxidant activity of MGN was determined by an ORAC assay, showing that the encapsulation of the drug was able to preserve its activity.

Instead, the present work wants to investigate, by in vitro assays, the capability of MGN–NLC to act as a potential drug delivery system for MGN ocular administration. In vitro assays were performed to evaluate the formulation cytotoxicity and its antioxidant role by mitigating ROS formation and GSH depletion induced by H_2_O_2._ Furthermore, MGN ocular permeability has been evaluated through an ex vivo test.

## 2. Materials and Methods

### 2.1. Materials

Compritol 888 ATO was obtained from Gattefossè (Milan, Italy), oil Miglyol 812 was provided by Eigenmann & Veronelli S.p.A. (Milan, Italy) and Lutrol F68 was purchased from BASF ChemTrade GmbH (Burgbernheim, Germany). Mangiferin, Phosphate Buffered Saline (PBS commercial 10×) and all solvents were purchased from Merck (Milan, Italy). Ultrapure water (resistivity > 18.2 MΩ·cm) was obtained by reverse osmosis (Milli-Q, Millipore Iberica, Madrid, Spain). The ARPE 19 cell line was purchased from ATCC, and bromide 3-(4,5-dimethylthiazol-2-yl)-2,5-diphenyltetrazolium (MTT), dimethyl sulfoxide (DMSO), sulfhydryl reagent 5,5′-dithio-bis (2-nitrobenzoic acid) (DTNB) and 2′,7′- dichlorofluorescein diacetate (DCFH-DA) were purchased from Sigma–Merk Life Science, Milan, IT.

### 2.2. MGN–NLC Formulation

MGN–NLC was formulated by high shear homogenization, followed by an ultrasound (HSH-US) method, according to our previous work [11]. The NLC was based on a lipid phase mixture made of Compritol 888 ATO (600 mg) and Miglyol 812 oil (400 mg). This melted lipid mixture (80 °C) containing MGN (0.1% *w*/*v*) was dispersed in hot surfactant solution (Lutrol F68 0.4% *w*/*v*; 80 °C) under homogenization at 17,500 rpm for 10 min. The obtained emulsion was ultrasonified (Labsonic 2000 B. Braun, Melsunen, Germany) for 8 min and then rapidly cooled by dilution with 25 mL of cold deionized water. The blank NLC was prepared following the same procedure, without MGN.

### 2.3. MGN–NLC Characterization

The Photon Correlation Spectroscopy (PCS) technique (Zetasizer Nano Series, Nano SP90, Malvern Instr., Malvern, UK)) was used to measure the particle size, polydispersity index (PDI) and zeta potential (ZP) of both unloaded and MGN-loaded NLC (diluted 10-fold with deionized water) [20]. Each measurement was carried out at least in triplicate at 20 °C and a 90° scattering angle. In order to study the stability of blank and MGN–NLC over time, each sample was characterized on the production day and after 1, 2, 3, 4, 8 and 12 weeks of storage at 25 °C [21].

To evaluate the morphology of MGN–NLC, Transmission Electron Microscopy (TEM; JEOL JEM-101) was used. Before observation, the NLC formulation was diluted 100-fold with deionized water and was then applied on the Formvar^®^-coated copper grid (TAAB Laboratories Equipment, Ltd., Aldermaston, UK) and dried [11].

### 2.4. Encapsulation Efficiency

The amount of encapsulated MGN in the nanoparticles was evaluated as previously reported [11]. Briefly, the MGN–NLC was diluted in H_2_O, filtered and freeze-dried overnight (Lio 5-P-Pascal, High Vacuum & Cryogenic System, Milan, Italy). A known amount of the obtained dry product was dissolved in methanol, and the MGN content was determined by UV spectrophotometry at 257 nm (T80^+^ UV/VIS Spectrometer, PG Instrument Ltd., Lutterworth, UK). Standard curves of the MGN were drawn over a concentration range of 0.5–10 µg/mL.

### 2.5. Lyophilization Stability

Before lyophilization, cryoprotectant (glucose or mannitol) at a concentration range of 0.5–5% (*w*/*v*) has been added to blank NLC (2 mL). Subsequently, the lyophilized powders were reconstituted to the original volume (2 mL) with deionized water for further PCS characterization.

### 2.6. Corneal Permeability Assay

Mangiferin corneal permeability was evaluated, in triplicate, following the protocol previously described by Varela-Garcia et al. [22]. Fresh bovine eyes (Compostelana de Carnes S. L., Santiago de Compostela, Spain) were immersed in PBS (PBS commercial, 10×) without antibiotics and transported, in an ice bath, to the laboratory. The corneas were then isolated, leaving a few millimeters of the surrounding sclera, and washed with PBS to be assembled in vertical diffusion Franz cells. Before starting the assay, the donor and receptor chambers were filled with a propylene glycol:water (40:60 *v*/*v*) solution and maintained at 37 °C with magnetic stirring to balance the tissues. After 1 h, the donor chambers were completely emptied and filled with 2 mL of MGN–NLC formulation. The experiment was carried out, protected from light to avoid MGN degradation and covering the donor chambers with parafilm to prevent evaporation. Samples of 1 mL were taken from the receptor chamber every 30 min for 6 h and replaced with the same volume of propylene glycol:water (40:60), removing any bubble from the diffusion cells. The samples were analyzed through HPLC (JASCO, Tokyo, Japan) equipped with a C18 column (Waters Symmetry C18, 5 µm, 4.6 × 250 mm) and ChromNAV software (ver.2, JASCO, Tokyo, Japan) at 254 nm. The analysis was carried out by isocratic elution of 0.1% formic acid:acetronitrile (87:13) at 1.5 mL/min and 26 °C. The injection volume was 100 µL, and the retention time was 12 min [23]. The standard solutions were 0.075–1 µg/mL of MGN in ethanol:propylenglycol:water (10:40:50). After 6 h of testing, the corneas were placed in Falcon tubes with 2 mL acetonitrile for 24 h at 37 °C to be later treated following a previously described protocol [24]. The MGN content in the samples was calculated from a previously validated calibration curve and expressed as cumulative mass permeated (µg) and cumulative mass permeated per surface (µg/cm^2^), considering a permeability area of 0.785 cm^2^. The MGN remaining in the donor chambers and accumulated in the corneas was also quantified. Finally, the steady state flux (J) and the lag time (t_lag_) were also calculated as described elsewhere [25].

### 2.7. Cell Culture and Treatments

In the present study, we investigated antioxidants’ effects on ARPE-19, a retinal pigment epithelial cell line, derived from the normal eyes of a 19-year-old male. The loss of retinal pigment epithelial cells is one of the leading causes of several eye diseases [26,27,28]. Briefly, ARPE-19 cells were trypsinized (0.25% trypsin-EDTA solution), counted and resuspended in DMEM F-12 30-2006^TM^ medium containing 2.5 mM L-glutamine, 15 mM Hepes, 0.5 mM sodium pyruvate and 1200 mg/L sodium bicarbonate. The cells were initially treated with blank NLC and MGN–NLC (MGN 1 mg/mL) at different concentrations (5, 10, 20, 40, 80, 100 and 150 µL/mL) for 24 h to identify the best concentration. Subsequently, the cells were seeded in six-well plates at a density of 3 × 10^5^ cells per well. The plates were then incubated at 37 °C and 5% CO_2_. After 24 h of seeding, insult was provoked with hydrogen peroxide (H_2_O_2_) at a concentration of 0.5 mM for 30 min. After 30 min, the solution containing hydrogen peroxide was removed, and fresh medium was added to each well. The experiment was performed as follows: untreated, 20 µL/mL MGN (standard Mangiferin), blank NLC 20 µL/mL (empty nanoparticles), 20 µL/mL MGN–NLC (standard MGN 20 mg/mL). After 24 h of treatment, 100 µL of the conditioned medium was taken for ROS measurement.

### 2.8. Cell Viability Assay

In order to evaluate the cytotoxic effect of blank NLC and MGN–NLC on the ARPE-19 cell line, the cells were cultured in 96-well plates (10.0 × 10^3^ cells/well in 100 µL of culture medium). Following 24 h treatments, 100 µL of a 0.25 mg/mL solution of 3-(4,5-dimethylthiazol-2-yl)-2,5-diphenyltetrazolium bromide (MTT) (ACROS Organics BV) was added to each well, and the cells were incubated for 2 h at 37 °C and 5% CO_2_. Then, the supernatant was removed, and, in order to dissolve the formazan salts produced by the active mitochondria, 100 µL of DMSO was added to each well. Finally, absorbance (OD) was measured in a microplate reader (Biotek Synergy-HT, Winooski, VT, USA) at λ = 570 nm. The amount of formazan was proportional to the number of viable cells in the sample. At least three separate experiments were conducted, with eight replicates per group.

### 2.9. GSH Assay

The intracellular content of reduced glutathione (GSH) was obtained, measuring the reaction of thiol groups with 5,5-dithio-bis-nitrobenzoic acid (DTNB) to form the yellow derivative 5′-thio-2-nitrobenzoic acid (TNB), measurable at λ = 412 nm (εM = 13,600 M^−1^·cm^−1^, where εM is a wavelength-dependent molar absorptivity coefficient). The measurements were performed in triplicate [29,30]. Briefly, the cells were cultured in T25, and afterwards, the treatments were trypsinized, harvested and centrifuged at 1500 rpm for 5 min at 4 °C. Then, the cell pellets were lysed by sonication and subsequently centrifuged at 12,000 rpm for 15 min at 4 °C. Lastly, 20 µL of the supernatant was added to the DTNB solution; after waiting for 20 min, the solution was read in a spectrophotometer at a wavelength of λ = 412 nm.

### 2.10. ROS Measurement

In order to measure ROS production on ARPE-19, a simple cell assay using 2′,7′- dichlorofluorescein diacetate (DCFH-DA) was performed. The fluorescence was analyzed by a fluorimeter by calculating the total H_2_O_2_ equivalents formed with respect to a standard curve obtained with increasing concentrations of H_2_O_2_. The experiments were conducted in triplicate.

### 2.11. Statistical Analysis

In order to perform statistical analysis of the data, a one-way ANOVA [31] test was used to assess significant differences among groups. The statistical significance (*p* < 0.05) of the differences between the experimental groups was determined by the Tukey test for the analysis of multiple comparisons.

## 3. Results and Discussion

### 3.1. MGN–NLC Characterization

**Figure 2:**
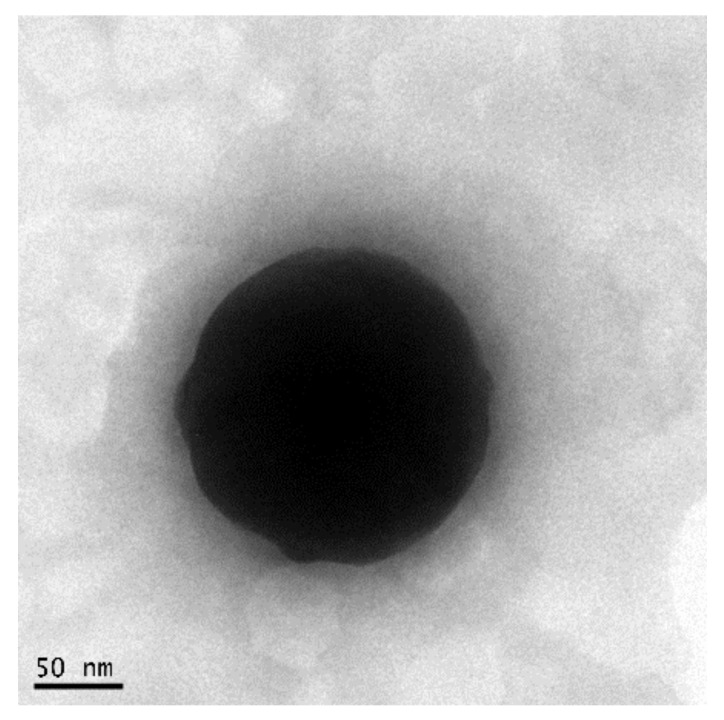
TEM image of MGN–NLC. The scale bar represents 50 nm. The sample size in the picture is 187.3 nm.

Blank and MGN–NLC were formulated by high shear homogenization, followed by the ultrasound (HSH-US) method, according to our previous work [11]. This method has been found to be highly reproducible and suitable for ocular applications due to the use of biocompatible excipients (Compritol 888 ATO solid lipid, Miglyol 812 oil and Lutrol F68 surfactant) [14,15] and a particle size below 200 nm [11]. The PCS data showed that the blank NLC had a polydispersity index (PDI) of 0.18 ± 0.1, a zeta potential (ZP) value of -28.6 ± 0.3 mV and a size below 200 nm (123.1 ± 0.1 nm), while MGN–NLC had a PDI value of 0.21 ± 0.02, a ZP of −23.5 mV and a size of 148.9 ± 0.1 nm. The latter was further confirmed by TEM (Figure 2), which showed that the MGN–NLC formulation was well structured and suitable for ocular administration. Moreover, the encapsulated MGN in the nanoparticles, evaluated by UV/VIS spectrometry, was approximately 92% [11]. Many challenges have been identified as bottlenecks during the development of lipid-based nanocarriers, and the most critical hurdle is probably the stability of the formulation.

**Figure 3:**
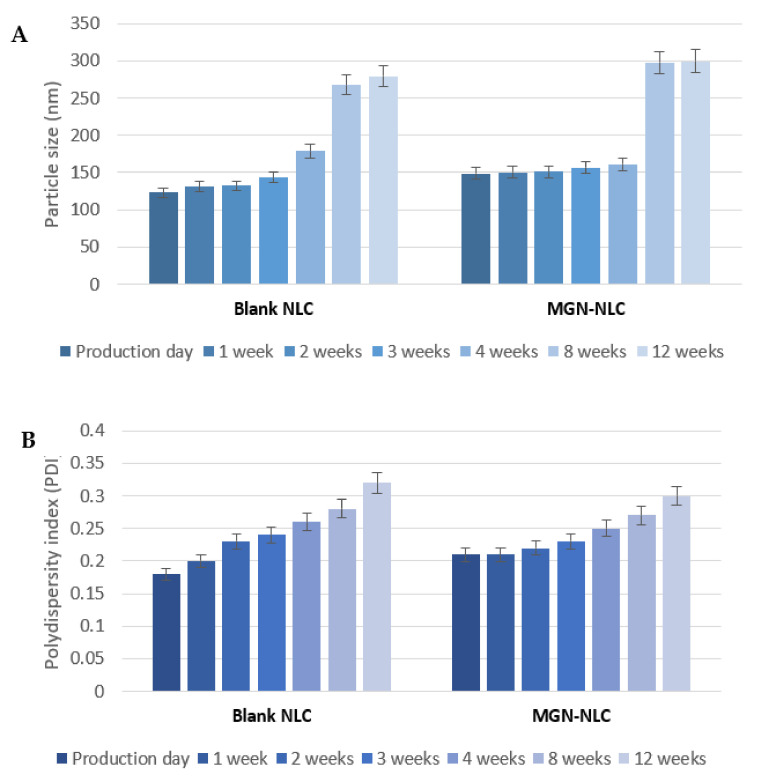

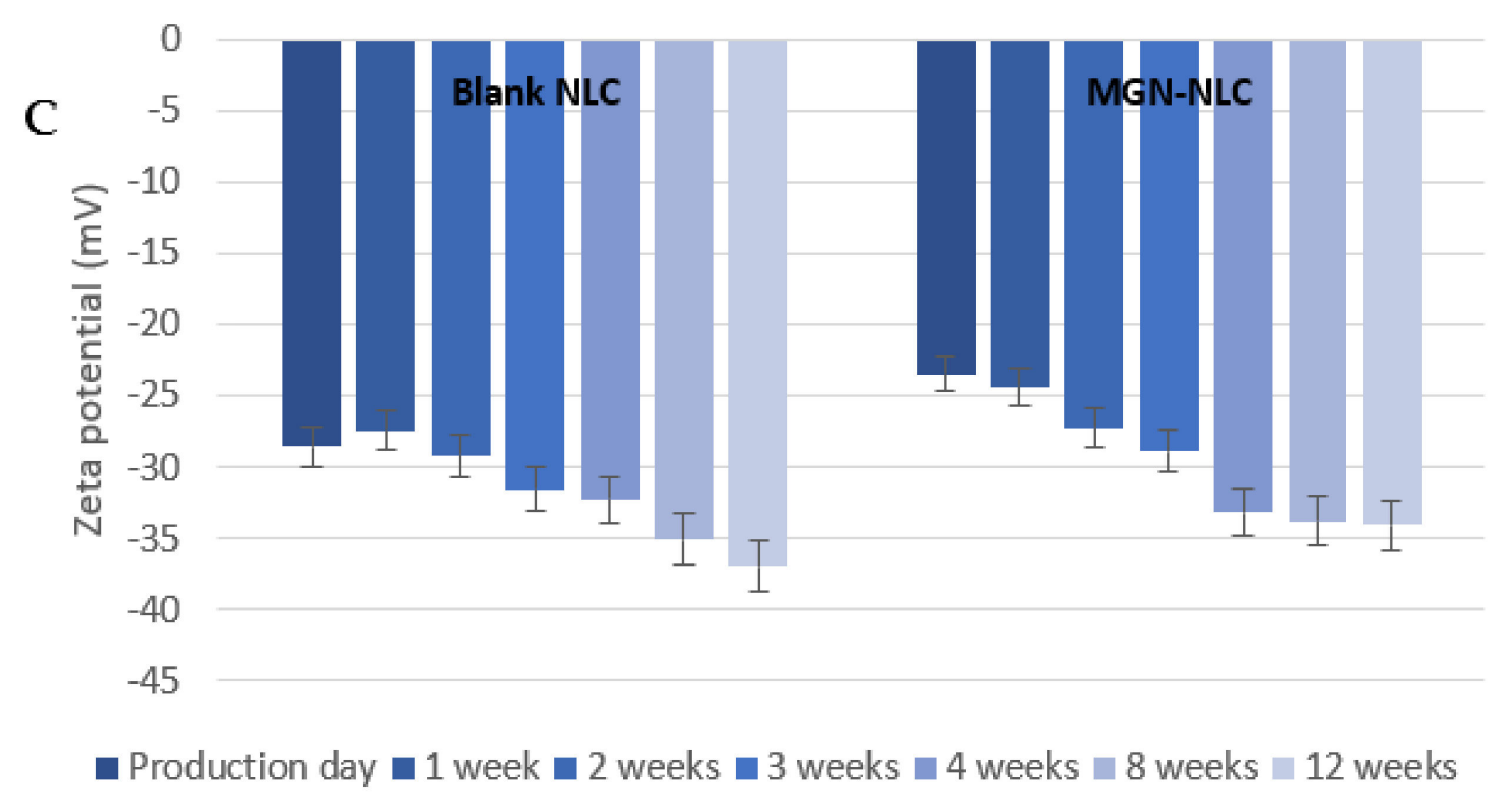
(**A**) Particle size, (**B**) polydispersity index (PDI) and (**C**) Z-potential of blank and MGN–NLC during 12 weeks of storage at 25 °C.

To investigate the stability of the formulations, nanotechnological parameters were monitored during 12 weeks of storage at 25 °C in an aqueous suspension. As demonstrated by PCS measurements, the formulation showed an acceptable stability after 4 weeks of storage; in particular, after this period, a significant difference was observed in terms of particle size and PDI and ZP values (Figure 3). As reported in our previous work [15], the excellent stability of the blank formulation during the first 2 weeks of storage was further confirmed by Turbiscan analysis.

### 3.2. Lyophilization Effects

**Figure 4:**
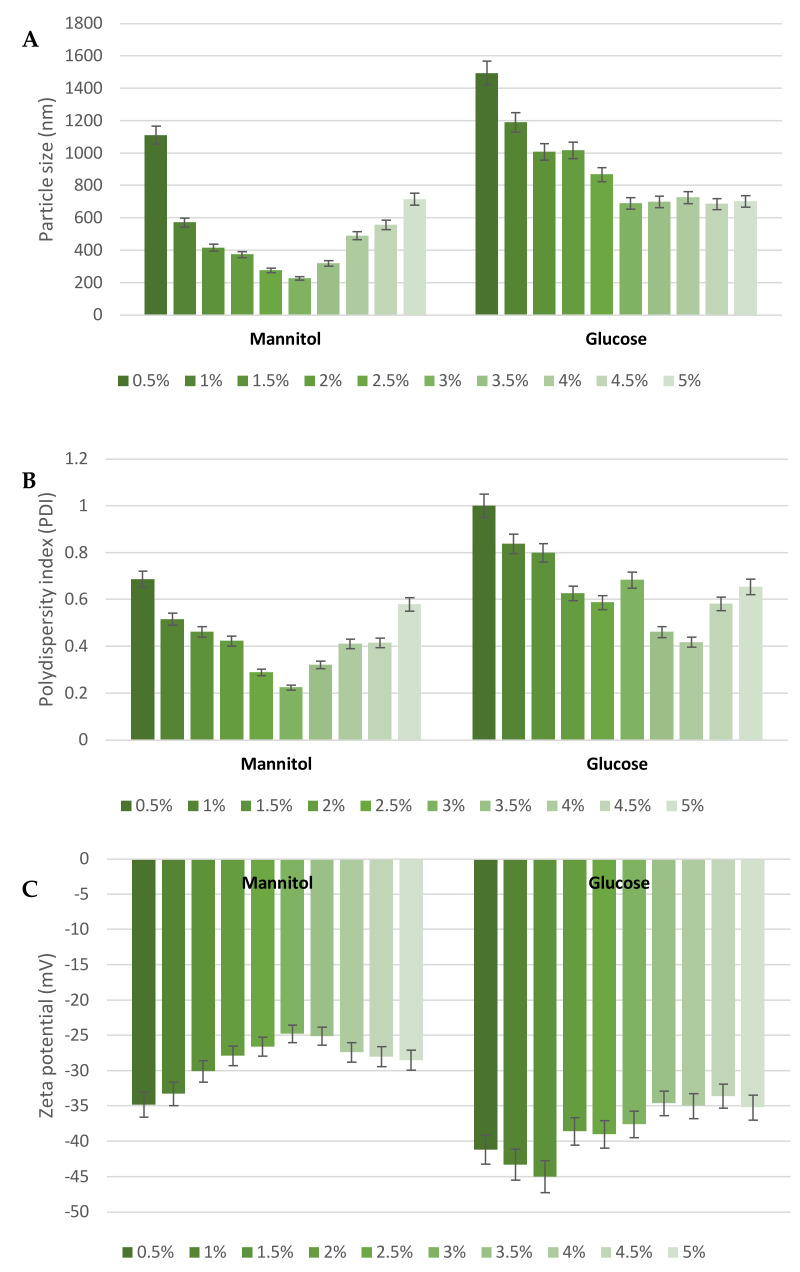
(**A**) Particle size, (**B**) polydispersity index (PDI) and (**C**) Z-potential of MGN–NLC using increasing concentrations of cryoprotectants (mannitol and glucose).

As reported above, the blank and MGN–NLC were stable for 4 weeks of storage in an aqueous suspension at 25 °C, but further stability was not achieved, probably due to physical and chemical phenomena, such as nanoparticles aggregation or microbiological contamination. The removal of a high amount of water (70–95%) in the lipid nanoparticle suspension is often the key to achieving maximum physical and chemical stability. The freeze-drying technique offers many advantages for this aim, and it is widely used to convert lipid nanocarriers into a powder of good physical stability. Therefore, we carried out the freeze-drying of the NLC formulation, and we also tested the stability of the system after reconstitution. Since this is a complex process that could destabilize nanoparticle systems, the addition of a cryoprotectant (i.e., glucose, sucrose or mannitol) is highly recommended. The evidence reported in the literature has shown that mannitol and glucose are the most used cryoprotectants for the freeze-drying of lipid nanoparticles [32,33]. Therefore, we tested both cryoprotectants (glucose and mannitol) at different concentrations (0.5–5% *w*/*v*) to identify the most effective one in protecting the lipid nanoparticles during freeze-drying. The obtained results demonstrated that glucose was not suitable for these formulations; in fact, all reconstituted samples showed a significant increase in particle size, ranging from 685.4 to 1494 nm, PDI values (approximately 0.6) and zeta potential (ZP) (around −38 mV). In contrast, acceptable results were observed using mannitol as a cryoprotectant. According to the literature [34], the addition of mannitol at 3% (*w*/*v*) represented the best concentration for obtaining an effective cryoprotectant effect (225.8 nm, PDI of 0.224 and ZP of −24.8 mV). This evidence demonstrated that the stabilization effect of cryoprotectants depends on their concentration and that, generally speaking, an increase in concentration beyond a certain level has a destabilizing effect on the nanoparticle suspension. In Figure 4, we reported the results regarding MGN–NLC, as those for blank NLC were similar.

### 3.3. Corneal Permeability Assay

**Figure 5:**
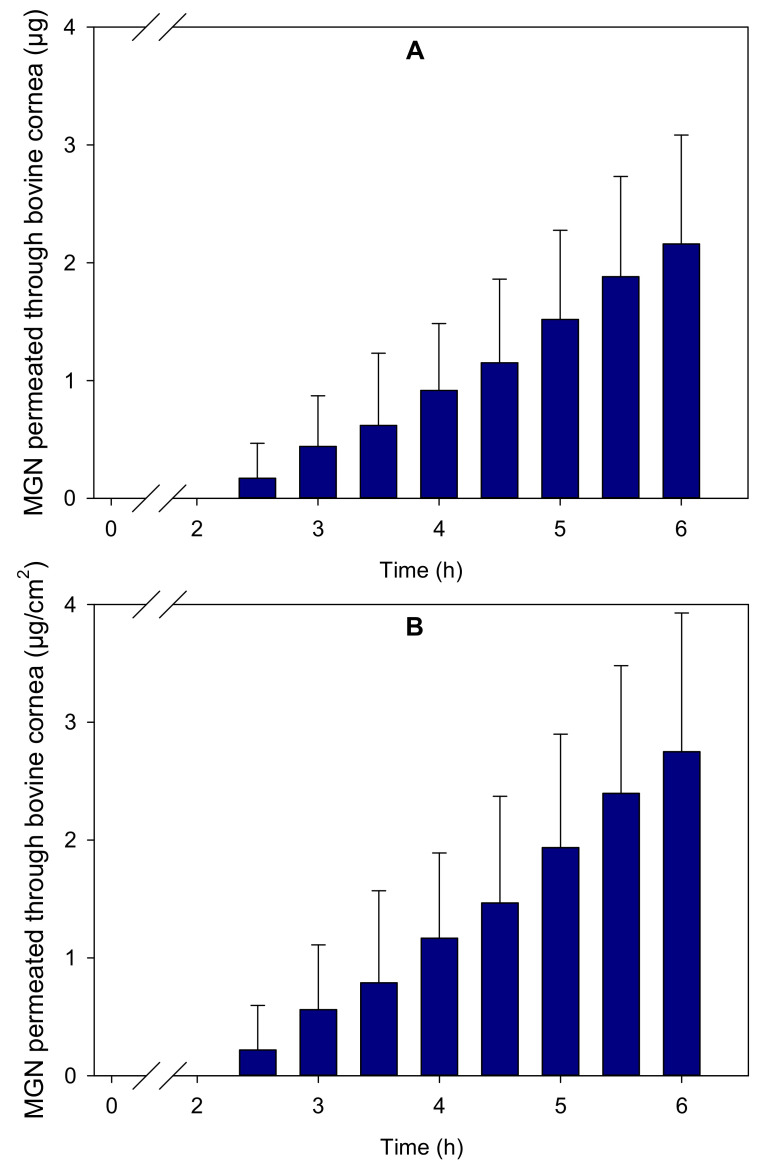
Accumulated amounts of MGN that permeated through bovine cornea for 6 h, expressed as (**A**) µg and (**B**) µg/cm^2^.

A bovine corneal permeability test for MGN in the MGN–NLC formulation was carried out with a view to ascertain the capability of MGN to accumulate into and permeate through the cornea towards the receptor medium, mimicking the aqueous humor. The amounts of MGN that remained in the donor chamber, permeated into the cornea and diffused to the receptor chamber were monitored. Propylene glycol:water (40:60) was used as the receptor medium [35] due to the low solubility of MGN in aqueous solutions [36]. The accumulative amount of MGN that permeated per area after 6 h was 2.75 (s.d. 1.18) µg/cm^2^, as reported in Figure 5, and 1.86 (s.d. 0.51) µg/cm^2^ of MGN was held in the cornea. A total of 47.86 (s.d. 2.5) µg/mL of MGN remained in the donor chamber. This concentration value, along with the steady state flux (J), which was estimated to be 0.73 µg/cm^2^/h, was used to calculate the permeability coefficient (P_app_) as 4.23 × 10^−6^ cm s^−1^. Furthermore, the t_lag_ was estimated to be 1.69 h.

The benefits of encapsulating MGN in NLC were evaluated in terms of the capability to promote ocular permeability through the cornea. A lag time of about 2 h was observed, in agreement with reports for other drugs [25,37], which is understandable considering the compact layered structure of the cornea. After 6 h of contact, a relevant amount of MGN was accumulated in the cornea (1.86 (s.d. 0.51) µg/cm^2^), and a larger amount was permeated (2.75 (s.d. 1.18) µg/cm^2^). The permeability coefficient (P_app_) obtained (4.23 × 10^−6^ cm s^−1^) was in the range of those recorded for other NLC [12] and for other hydrophobic molecules formulated as micelles [22,35].

### 3.4. Effect of Blank NLC and MGN–NLC on Cell Viability

**Figure 6:**
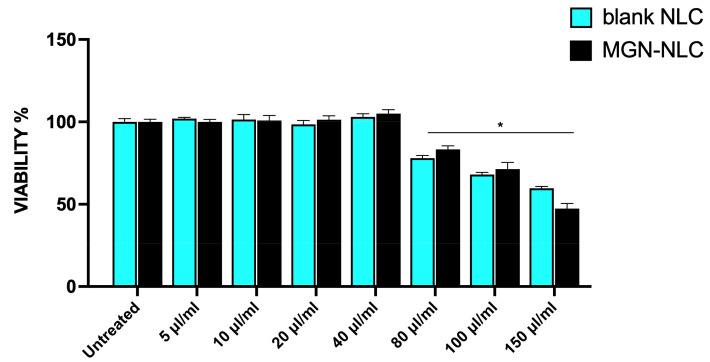
The viability assay in ARPE-19 cells measured by bromide 3-(4,5-dimethylthiazol-2-yl)-2,5-diphenyltetrazolium (MTT) assay. * *p* < 0.5 versus untreated.

Previously, studies have reported antioxidant and antinflammatory properties for mangiferin [29,38]. In order to assess the eventual toxicity of MGN–NLC and blank NLC compared to MGN treatment on ARPE-19, an MTT assay was performed (Figure 6). Cells were treated with different concentrations of blank NLC and MGN–NLC (from 5 μL/mL to 150 μL/mL). With the only exception of the highest concentration for both treatments (>80 μL/mL), no toxicity compared to the control was observed for all formulations (Figure 6). The data confirm that nanostructured lipid carriers at the same concentrations as those of mangiferin used in previous studies show no cytotoxicity effects alone or loaded with MGN [9,29,38].

### 3.5. MGN–NLC Mitigates ROS Production

**Figure 7:**
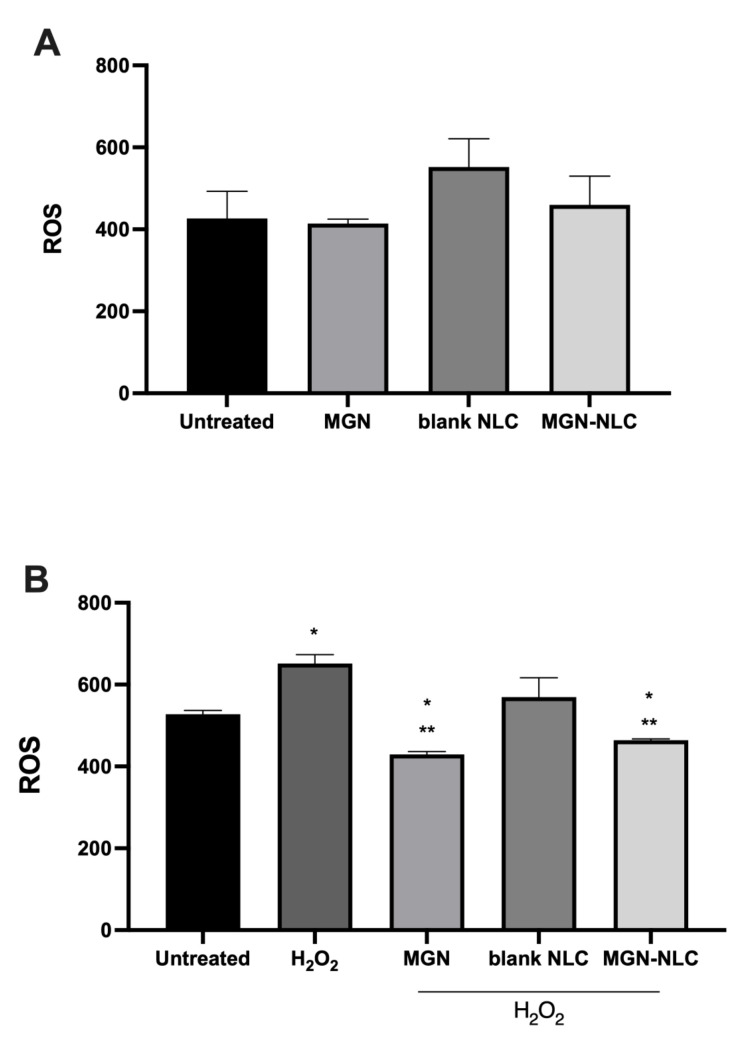
ROS production in ARPE-19, as measured by a simple cell assay using 2′,7′- Dichlorofluorescein diacetate (DCFH-DA). (**A**) ROS production in untreated cells and after treatments with 20 mg/mL MGN (standard mangiferin), blank NLC 20 µL/mL (empty nanoparticles) and 20 µL/mL MGN–NLC (standard MGN 20 mg/mL). (**B**) Cells were treated with hydrogen peroxide (H_2_O_2_) at a concentration of 0.5 mM. After 30 min, the solution containing hydrogen peroxide was removed, fresh medium was added to each well and treatments with 20 mg/mL MGN (standard mangiferin), blank NLC 20 µL/mL (empty nanoparticles) and 20 µL/mL MGN–NLC (standard MGN 20 mg/mL) were performed. * *p* < 0.05 versus untreated; ** *p* < 0.05 versus H_2_O_2_.

The antioxidant properties of polyphenols or natural substances are often related to a decrease in ROS formation in the tested cells. The presence of ROS reflects the induction of oxidative stress and is responsible for the onset of cardiovascular, metabolic and neoplastic diseases, as well as macular degeneration [9,28,39,40,41]. To investigate a possible maintenance of the protective role of MGN in oxidative stress, we treated ARPE-19 in the presence and absence of 0.5 mM H_2_O_2_. We showed in Figure 7a that free MGN, blank NLC and MGN–NLC showed no significant changes in ROS production, while free MGN and MGN–NLC treatments were similarly able to mitigate the induced ROS formation (Figure 7b). The obtained results show that mangiferin delivered by NLC maintains its antioxidant and radical scavenger properties, which suggests that the delivery of mangiferin through nanostructured lipid carriers does not alter the antioxidant power of the molecule.

### 3.6. MGN–NLC Maintains Redox Balance by Increasing GSH Levels

**Figure 8:**
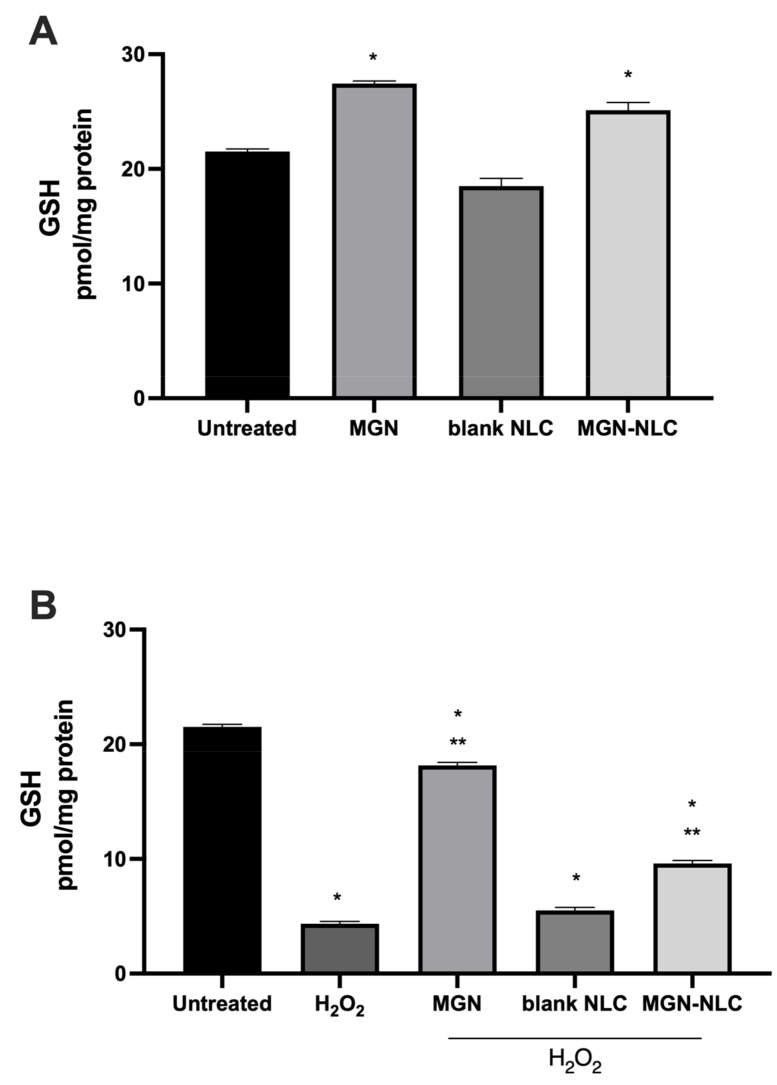
Intracellular content of reduced glutathione (GSH). (**A**) GSH level under normal conditions, (**B**) GSH level following 0.5 mM H_2_O_2_ treatment for 30 min. * *p* < 0.05 versus untreated; ** *p* < 0.05 versus H_2_O_2_.

Consistent with the studies demonstrating that mangiferin induces the cellular endogenous antioxidant system, we evaluated the levels of reduced glutathione (GSH) present in the cells following the treatments [42,43]. Both MGN and MGN–NLC were able to increase GSH basal levels compared to the control, as shown in Figure 8. The treatment with free MGN and MGN–NLC was able to mitigate the glutathione depletion caused by H_2_O_2_, confirming the maintenance of the oxidative stress role for MGN–NLC. The results clearly show that MGN–NLC is able to induce GSH levels, confirming the preservation of MGN effects within the nanostructures.

## 4. Conclusions

The growing interest in natural compounds motivated us to investigate the development of new drug delivery systems capable of increasing the ocular bioavailability of MGN, a promising antioxidant for the treatment of different oxidative stress-related ocular diseases. As previously demonstrated by our group, the encapsulation of MGN in nanostructured lipid carriers (MGN–NLC) showed good nanotechnological parameters suitable for ocular delivery. So, in the present work, the capability of NLC to act as a potential drug delivery system for MGN ocular administration was evaluated through in vitro and ex vivo assays. No cytotoxic effects were observed for both unloaded and MGN-loaded NLC, in vitro, using retinal pigment epithelium cells (ARPE-19). Moreover, the capability of the NLC to maintain the antioxidant role of MGN was also demonstrated in vitro by mitigating ROS formation and GSH depletion induced by H_2_O_2_. Although more investigations are needed to obtain more details and to further confirm the evidence provided so far, all this evidence suggests a potential application of MGN–NLC in the treatment of ocular diseases associated with marked oxidative stress.

## Data Availability

Data is available on the request from the corresponding author.
